# Evaluation of calculation processes of apparent diffusion coefficient subtraction method (ASM) imaging

**DOI:** 10.1371/journal.pone.0282462

**Published:** 2023-02-27

**Authors:** Majd Barham, Masahiro Kuroda, Yuuki Yoshimura, Kentaro Hamada, Abdullah Khasawneh, Kohei Sugimoto, Kohei Konishi, Nouha Tekiki, Irfan Sugianto, Babatunde O. Bamgbose, Hinata Ishizaka, Yudai Shimizu, Yuki Nakamitsu, Wlla E. Al-Hammad, Ryo Kamizaki, Akira Kurozumi, Toshi Matsushita, Seiichiro Ohno, Junichi Asaumi

**Affiliations:** 1 Department of Oral and Maxillofacial Radiology, Okayama University Graduate School of Medicine, Dentistry and Pharmaceutical Sciences, Okayama, Japan; 2 Radiological Technology, Graduate School of Health Sciences, Okayama University, Okayama, Japan; 3 Radiology Diagnosis, Okayama Saiseikai General Hospital, Okayama, Japan; 4 Central Division of Radiology, Okayama University Hospital, Okayama, Japan; Institute of Biophysics, China Academy of Sciences, CHINA

## Abstract

A number of restricted diffusion (RD) imaging techniques, such as diffusion kurtosis (DK) imaging and Q space imaging, have been developed and proven to be useful for the diagnosis of diseases, including cerebral gliomas and cerebrovascular infarction. In particular, apparent diffusion coefficient (ADC) subtraction method (ASM) imaging has become available recently as a novel RD imaging technique. ASM is based on the difference between the ADC values in an image pair of two ADC maps, ADC basic (ADC_b_) and ADC modify (ADC_m_), which are created from diffusion-weighted images taken using short and long effective diffusion times, respectively. The present study aimed to assess the potential of different types of ASM imaging by comparing them with DK imaging which is the gold-standard RD imaging technique. In the present basic study using both polyethylene glycol phantom and cell-containing bio-phantom, three different types of ASM images were created using different calculation processes. ASM/A is an image calculated by dividing the absolute difference between ADC_b_ and ADC_m_ by ADC_b_ several times. By contrast, ASM/S is an image created by dividing the absolute difference between ADC_b_ and ADC_m_ by the standard deviation of ADC_b_ several times. As for positive ASM/A image (PASM/A), the positive image, which was resultant after subtracting ADC_b_ from ADC_m_, was divided by ADC_b_ several times. A comparison was made between the types of ASM and DK images. The results showed the same tendency between ASM/A in addition to both ASM/S and PASM/A. By increasing the number of divisions by ADC_b_ from three to five times, ASM/A images transformed from DK-mimicking to more RD-sensitive images compared with DK images. These observations suggest that ASM/A images may prove useful for future clinical applications in RD imaging protocols for the diagnosis of diseases.

## Introduction

Diffusion-weighted (DW) imaging is extensively used in clinical practice as a non-invasive magnetic resonance (MR) imaging technique [1]. The apparent diffusion coefficient (ADC) map, which is an image derived from DW images, has been widely applied in the clinical setting [2]. Mechanistically, the ADC map reflects the diffusion of water molecules within tissues [3]. A number of restricted diffusion (RD) imaging techniques, such as diffusion kurtosis (DK) imaging and Q space imaging [4], have been developed. DK imaging is one of the RD imaging techniques that is based on the quantification of the deviation of water molecule diffusion from the Gaussian distribution of unrestricted diffusion [5, 6]. RD represents the movement of water molecules in the presence of barriers, including cell membranes and intracellular organelles [7]. The DK imaging technique has been successfully applied for the detection of diseases, such as cerebral gliomas and cerebrovascular infarction [8, 9].

Recently, a novel RD imaging technique named ADC subtraction method (ASM) was developed, which was created basically using the difference between two ADC values taken using two different diffusion times [10]. To create ASM images for clinical trial testing, an ASM imaging software was created in-house based on the principle of ASM [11]. A fast imaging sequence was developed for the clinical application of ASM [12]. In addition, special MR phantom using polyethylene glycol (PEG) has been developed and proved to be the standard for RD imaging, such as ASM and DK imaging [13].

The purpose of the present study was to assess the potential of different types of ASM imaging as a new RD imaging technique by comparing it with DK imaging. In addition, the secondary aim was to identify a type of ASM imaging that shares similarities with DK imaging and another type that is potentially more sensitive for RD than DK imaging. The present study compared an array of ASM images created using different calculation processes from both PEG phantom and cell-containing bio-phantom [14] before finally evaluating the effects of these processes on ASM images.

## Materials and methods

### Phantoms

Two types of phantoms were prepared, bio-phantom and PEG phantom. For bio-phantom, Jurkat cells were purchased from the RIKEN BioResource Center (https://web.brc.riken.jp/en/). For cell culture, 10% FBS (Filtron Pty Ltd.) and 1% penicillin-streptomycin-neomycin (Gibco; Thermo Fisher Scientific, Inc.) were added to RPMI 1640 medium (pH 7.4; Gibco; Thermo Fisher Scientific, Inc.). The incubation was performed at 37°C with 5% CO_2_. The number of cells with a diameter >8 μm was counted using an electric cell counter (Beckman Coulter, Inc.) prior to bio-phantom preparation [14, 15]. Jurkat cells were encapsulated in bio-phantoms made of Phytagel^™^ (cat. no. P-8169; Sigma-Aldrich; Merck KGaA). The following two types of bio-phantoms were prepared: A pellet-like high-cellularity (HC) phantom and a low-cellularity (LC) phantom fixed with Phytagel^™^. After preparing the phantoms, they were placed in a microcuvette (Halbmikro 1.5 ml; Greiner Bio-One International GmbH).

The PEG phantom [16], which was used as the newly developed RD phantom [13], consisted of the following three components: i) PEG (cat. no. P3640-500G; Sigma-Aldrich; Merck KGaA) as a diffusion modifier; ii) NaN_3_ (Katayama Chemical Industry Co., Ltd.) as an antiseptic; and iii) double distilled water. This phantom solution was heated and diluted using double distilled water to achieve concentrations of 40, 80 and 120 mM with 0.03% w/w NaN_3_. After preparing the phantoms, they were placed in a microcuvette (Halbmikro 1.5 ml).

### Preparation for MR imaging

After preparing the microcuvettes containing bio-phantom and PEG phantom, they were installed in a phantom container that had an outer diameter of 9.5 cm in length, 14 cm in width and 7 cm in height [17]. The interior of the container was filled with physiological saline (PS; 0.9% NaCl). The phantom container was then placed in a self-constructed bio-phantom heating device, which was made of ethylene-vinyl acetate copolymer and was attached to a circulating thermostatic chamber (BF-41 Thermo-Mate; Yamato Scientific Co., Ltd.). The temperature of the bio-phantom was adjusted to ~37°C, similar to that in the human body. During MR imaging, an optical fiber thermometer (Fluoroptic^™^ m3300; LumaSense Technologies Inc.) was installed in the microcuvette for real-time phantom temperature measurements.

### MR imaging of DW images

A 3.0T MRI device (MAGNETOM Prisma VE11C; Siemens AG) was used which had a 20-channel head/neck coil. Table 1 provides a list of the imaging parameters used to produce the DW images for DK imaging and ASM. For DK imaging, single shot-echo planar imaging (SS-EPI) was used in three sequences of DKI-1, DKI-2 and DKI-3 (Table 1), based on Tim Trio (long) protocol, (https://medicine.musc.edu/-/sm/medicine/departments/f/tim-trio-long.ashx?la=en), which is one of the recommended DKI protocols for Siemens scanners. This protocol requires 30 independent diffusion gradient directions and the deactivation of diffusion tensor processing. Moreover, it requires obtaining the data in two blocks; main block with three b-values and a secondary block with nine additional images of b-value 0. To improve resolution, two identical DK image data (DKI-1 and DKI-2) were acquired using the same acquisition parameters, averaged and used for the main block [18]. For ASM, two types of readout segmentation of long variable echo-train (RESOLVE) sequences were used: RESOLVE-basic and RESOLVE-modify. These were obtained for ASM by using different b-values. For RESOLVE-basic, the b-values were set to the following three points: 0, 500 and 1,000 sec/mm^2^. For RESOLVE-modify, the b-values were set to the following four points: 0, 500, 1,000 and 10,000 sec/mm^2^. Because the number of b-values was different, the δ, which represents the motion probing gradient (MPG) pulse duration and Δ, representing the MPG pulse spacing, of both sequences changed. In the following formula used to calculate b-values (Eq 1),

$$\text{b}=\gamma^{2}{\text{G}}^{2}\delta^{2}\;\text{x}\;(\Delta -\delta /3)$$ (1)

the term ‘Δ − δ/3’ is called the effective diffusion time (EDT) and represents the time during which diffusion phenomena are detected. In addition, γ is the gyromagnetic ratio of protons and G is the gradient magnetic field strength. The addition of the b-value of 10,000 for RESOLVE-modify results in prolonging δ and Δ, which in turn prolongs EDT of DW images with b-values 0, 500 and 1000. EDT of RESOLVE-basic and RESOLVE-modify were 39.3 and 46.0 msec, respectively. EDT of this modification sequence is elongated up to the maximum limit of EDT. DW imaging for the ADC map, ASM and DK images was performed five times for the HC bio-phantom and nine times for LC bio-phantom. For imaging the PEG phantom, DW imaging was performed 60 times for obtaining the ADC map and ASM images and 45 times for DK images.

**Table 1:** Imaging characteristics of diffusion-weighted images for diffusion kurtosis imaging and apparent diffusion coefficient subtraction method.

Target imaging	DKI^1^		ASM^2^	
Sequences	DKI-1& DKI-2	DKI-3	RESOLVE^3^-basic	RESOLVE-modify
Parameters				
Phase direction	AP^4^	AP	AP	AP
Imaging time (min:sec)	6:24	1:12	13:28	19:06
Diffusion time (msec)	28.9	-	39.3	46.0
Diffusion direction	30	30	3	3
b-value (sec/mm^2^)	0,500,1000	0	0,500,1000	0,500,1000,10000
δ^5^ (msec)	13.8	-	5.6	15.6
Δ^6^ (msec)	33.5	-	41.2	51.2
TR^7^ (msec)	6000	6000	8000	8000
TE^8^ (msec)	75	75	86	106
ES^9^ (msec)	0.93	0.93	0.56	0.56
FOV^10^ (mm)	120	120	120	120
Segments	1	1	7	7
Slice number	5	5	1	1
Slice thickness (mm)	5	5	5	5
Matrix	82x82	82x82	224x224	224x224
BW^11^ (Hz/pixel)	1220	1220	399	399
Averages	1	9	2	2

^1^Diffusion kurtosis imaging

^2^Apparent diffusion coefficient subtraction method

^3^Readout segmentation of long variable echo-trains

^4^Antero-posterior

^5^Motion probing gradient (MPG) pulse duration

^6^MPG pulse spacing

^7^Repetition time

^8^Echo time

^9^Echo space

^10^Field of view

^11^Band width

### Creation of ADC maps

To create the ADC maps, the ADC value of each pixel was calculated using Eq 2 [19],

$$\text{ADC}=\text{In}\;({\text{S}}_{0}/\text{S})/\text{b}$$ (2)

where S is the signal intensity and S_0_ is the signal intensity when the b-value is 0 sec/mm^2^. Two ADC maps, ADC basic (ADC_b_) and ADC modify (ADC_m_), were created from the DW images acquired using RESOLVE-basic (short EDT of 39.3 msec) and RESOLVE-modify (long EDT of 46.0 msec) sequences, respectively, using three different b-values: 0, 500 and 1,000 sec/mm^2^. The purpose of using a b-value of 10,000 sec/mm^2^ was for preparing a long EDT-RESOLVE-modify sequence for three DW images of b-values 0, 500 and 1000. Therefore, the DW image of b-value 10,000 was not used in the calculation process of ASM.

### Creation of ASM images

**Fig 1:**
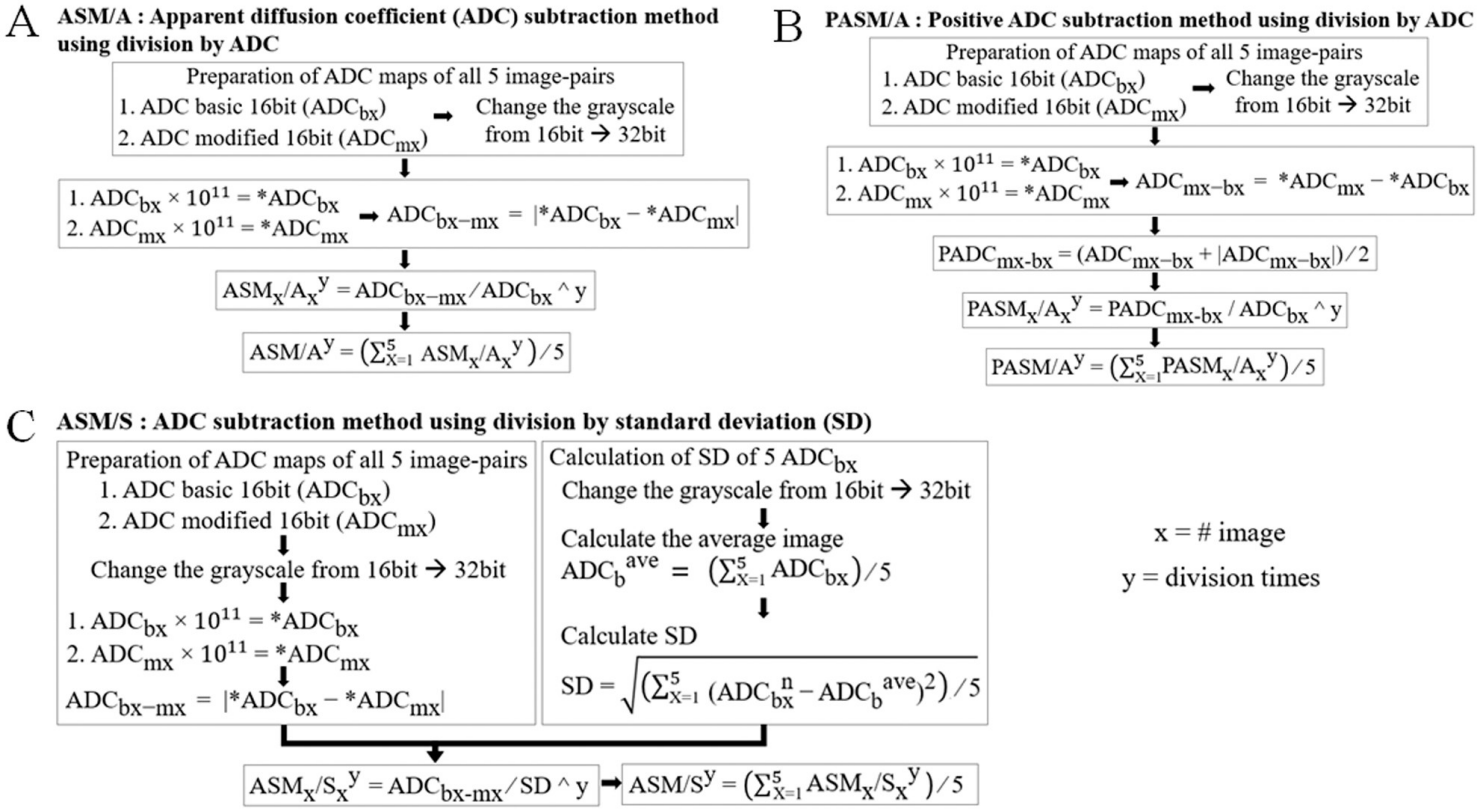
Calculation processes of the three types of ASM image. (A) ASM/A is an image indicated by the absolute difference between two ADC values, ADC_b_ and ADC_m_, divided by ADC_b_ ≤ five times. (B) PASM/A is an image indicated by the positive values of the difference between the two ADC values, which is deduced by subtracting ADC_b_ from ADC_m_, divided by the ADC_b_ value ≤ five times. (C) ASM/S is an image indicated by the absolute difference between the ADC values, ADC_b_ and ADC_m_, divided by the SD image. ADC, apparent diffusion coefficient; ASM, ADC subtraction method, ADC_b_, ADC basic; ADC_m_, ADC modify; PASM/A, positive ASM; SD, standard deviation; S, SD image.

In the present study, three types of ASM images, ASM/A, positive ASM/A (PASM/A) and ASM/S, were prepared using the ImageJ 1.52p software (National Institutes of Health) and an in-house software [11] following the calculation processes (Fig 1). Briefly, ASM images were created using the difference of ADC values in an image pair of two ADC maps (ADC_b_ and ADC_m_) after changing the grayscale of the ADC maps from 16 to 32 bits and multiplying each ADC map by a constant of 10^11^ to make the difference bigger for more accurate calculations. Typically, variations in ADC values increase when the ADC values are high. Therefore, to minimize this variation, the differences in ADC values in the two ADC maps should be modified by dividing it by the standard deviation (SD) of the ADC values or the ADC value itself.

To calculate the ASM/A, the ADC subtraction method was applied using division by the ADC map, where the absolute difference between the ADC values (ADC_b_ and ADC_m_) was divided by ADC_b_ ≤ five times.

As for PASM/A, the positive ADC subtraction method was applied instead by using division by the ADC map, where the difference between the ADC values was calculated by subtracting ADC_b_ from ADC_m_. When this difference resulted in negative values, they were substituted to zero values as shown in Fig 1B. Lastly, the positive values of the difference were divided by the ADC_b_ value ≤ five times.

Regarding ASM/S, the ADC subtraction method was applied by using division by the SD image, where the ASM/S was calculated by dividing the absolute difference between the ADC values (ADC_b_ and ADC_m_) by the SD image, which was created from five ADC_b_ maps using Eq 3,

$$\text{SD}=\sqrt{[\sum_{n=1}^{5}{{\text{(ADC}}_{\text{b}}{}^{n}-\;{\text{ADC}}_{\text{b}}{}^{ave})}^{2}]/5}$$ (3)

where ADC_b_^ave^ is the average ADC value from five ADC_b_ maps.

The processes of the three aforementioned types of ASM images were calculated for five image pairs. Thereafter, an average ASM image was calculated for each type.

### Creation of DK images

The DW images, obtained using sequences of DKI-1, DKI-2 and DKI-3, were processed using DKE version 2.6 (https://medicine.musc.edu/departments/centers/cbi/dki), which is a software tool for the post-processing of DK imaging datasets. This tool was then used to prepare a mean kurtosis (MK) image, which is a mean of kurtosis values calculated for each spatial direction. To create MK images, the MK value for each pixel was calculated using Eq 4 [13], according to the theoretical description of ‘DKI protocols’ (https://medicine.musc.edu/departments/centers/cbi/dki/protocols),

$$\text{MK}=[\text{In}\;(\text{S}/{\text{S}}_{0})+\text{b}\;\text{x}\;\text{ADC}]\;\text{x}\;6/({\text{b}}^{2}\;\text{x}\;{\text{ADC}}^{2})$$ (4)

where S is the signal intensity of pixels in the DW image and S_0_ is the signal intensity when the b-value is 0 sec/mm^2^. The b-values used were 0, 500 and 1,000 sec/mm^2^ for both DKI-1 and DKI-2 and only 0 sec/mm^2^ for DKI-3. As for the term used in this research, the term ‘DK image’ was used to indicate the MK image created.

### Statistical analysis

All results are presented as the mean ± standard deviation. Kruskal-Wallis test with Dunn’s method were performed using Bell Curve for Excel (Social Survey Research Information Co., Ltd) and SPSS software v27.0 (IBM Corp.) to compare the differences among the different values created in the present study. P<0.05 was considered to indicate a statistically significant difference. To examine the correlation between ADC_b_ and SD values, linear regression analysis was performed to calculate the R^2^-value using Bell Curve for Excel.

## Results

### Qualitative evaluation of the ADC map, DK image and ASM images

**Fig 2:**
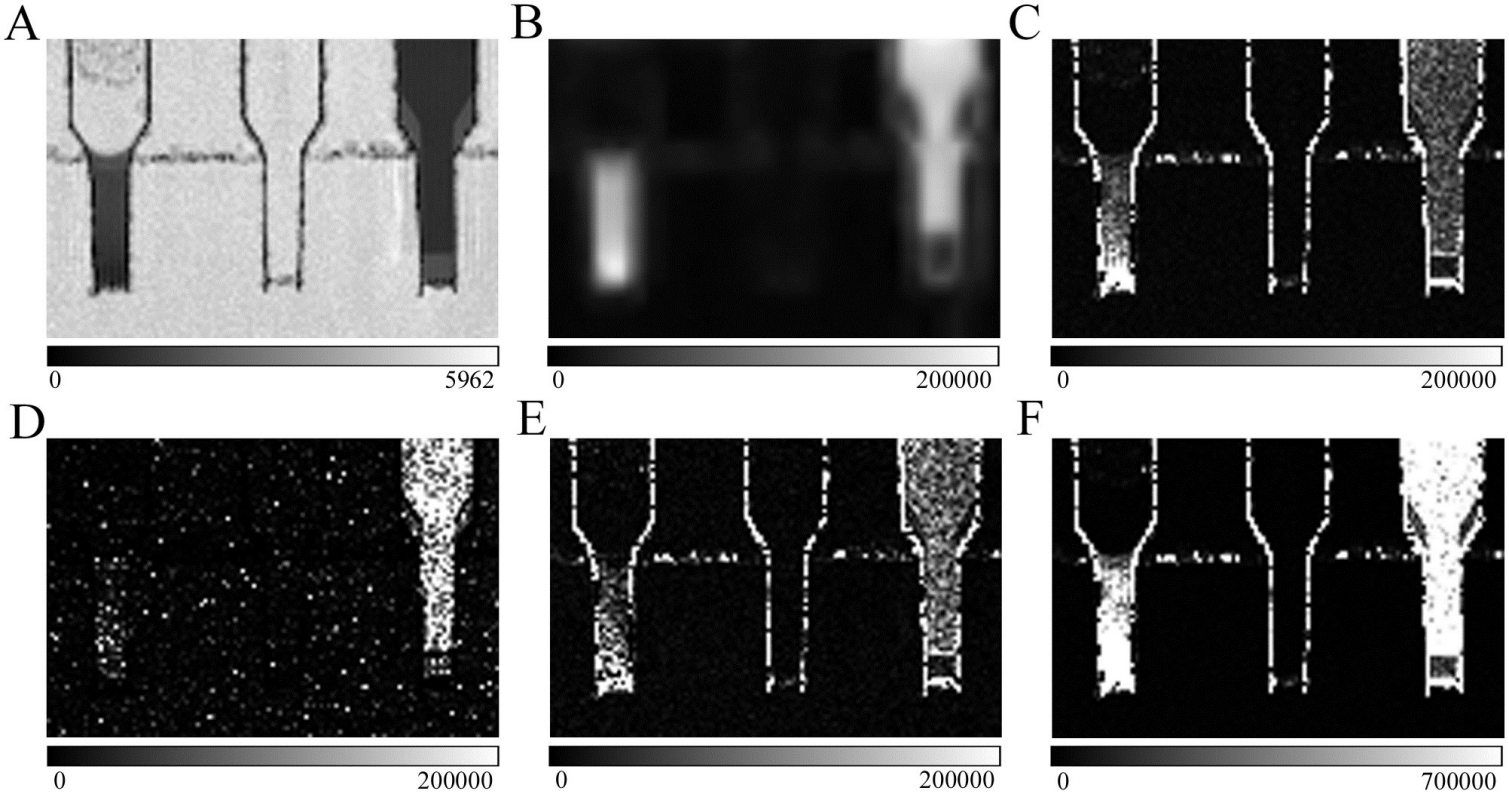
Comparison between several types of images for different phantoms. (A) ADC_b_. (B) DKI. (C) ASM/A^3^. (D) ASM/S^3^. (E) PASM/A^3^. (F) ASM/A^5^. From left to right in every image, phantoms are high-cellularity bio-phantom, physiological saline phantom and 120 mM polyethylene glycol phantom. ADC, apparent diffusion coefficient; ADC_b_, ADC basic; DKI, diffusion kurtosis image; ASM, ADC subtraction method; ASM/A^3^, ASM division by ADC_b_ three times; ASM/S^3^, ASM division by standard deviation image three times; PASM/A^3^, positive ASM division by ADC_b_ three times; ASM/A^5^, ASM division by ADC_b_ five times.

Fig 2 shows a comparison between several types of images for different phantoms. An opposite correlation is seen between the low-signal ADC map (Fig 2A) and the high-signal RD images including the DK (Fig 2B) and ASM images (Fig 2C–2F) for both the HC bio-phantom and the 120 mM PEG phantom. By contrast, the signals of ASM images showed the same tendency as those of the DK image, which is considered to be a gold standard for restricted imaging [20]. Furthermore, for comparison among the different types of ASM images, based on the number of divisions by the ADC_b_ map, it appeared that ASM/A^5^ (Fig 2F), which was the resultant image of the five-time division, showed the higher signal compared with that in ASM/A^3^ (Fig 2C) for both the HC bio-phantom and 120 mM PEG phantom. Based on the type of divisions, ASM/S^3^ (Fig 2D) showed the lower signal compared with that in ASM/A^3^ and ASM/A^5^ for the HC bio-phantom. For the 120 mM PEG phantom, ASM/S^3^ exhibited similar signals as ASM/A^5^ but higher signals compared with that in ASM/A^3^. In addition, comparing with the signals emitted by the original ASM/A^3^, PASM/A^3^ (Fig 2E) showed higher signals for the 120 mM PEG phantom and slightly lower signals for HC bio-phantom.

### ADC values of each phantom

**Fig 3:**
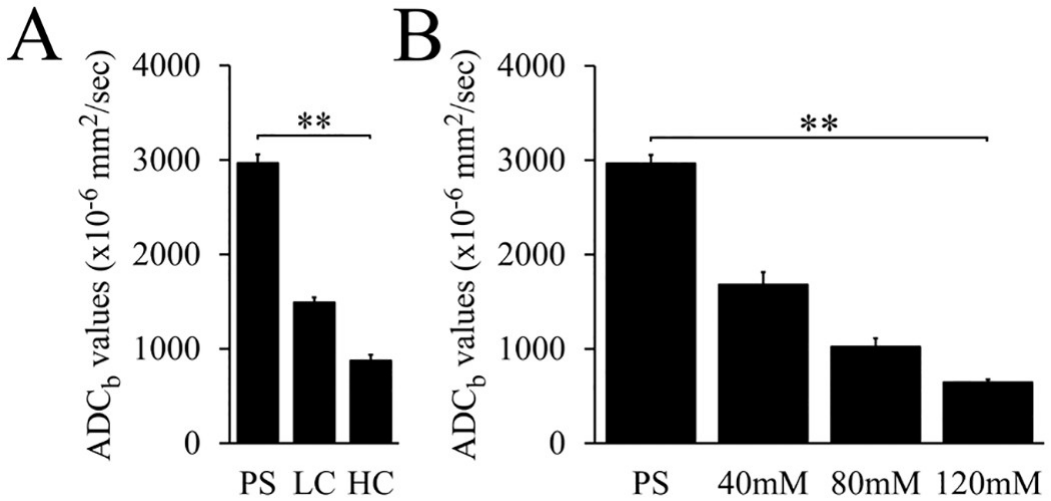
ADC values of different phantoms. (A) ADC_b_ values of bio-phantoms of Jurkat cells. (B) ADC_b_ values of polyethylene glycol phantoms. Error bar represents standard deviation for each value. **P<0.01, using Kruskal-Wallis test. PS, physiological saline; LC, low-cellularity; HC, high-cellularity; ADC, apparent diffusion coefficient; ADC_b_, ADC basic.

Fig 3A shows the ADC_b_ values of the bio-phantoms. As the cellularity of the bio-phantom increases, the ADC_b_ value decreases significantly (P<0.01). The mean and SD value of ADC_b_ of HC bio-phantom was (874±50) x 10^−6^ mm^2^/sec. Fig 3B shows the ADC_b_ values of PEG phantoms. As the concentration of PEG increases, ADC_b_ value decreases significantly (P<0.01). The mean and SD value of ADC_b_ of the 120 mM PEG phantom was (644±23) x 10^−6^ mm^2^/sec.

### SD values of each phantom

**Fig 4:**
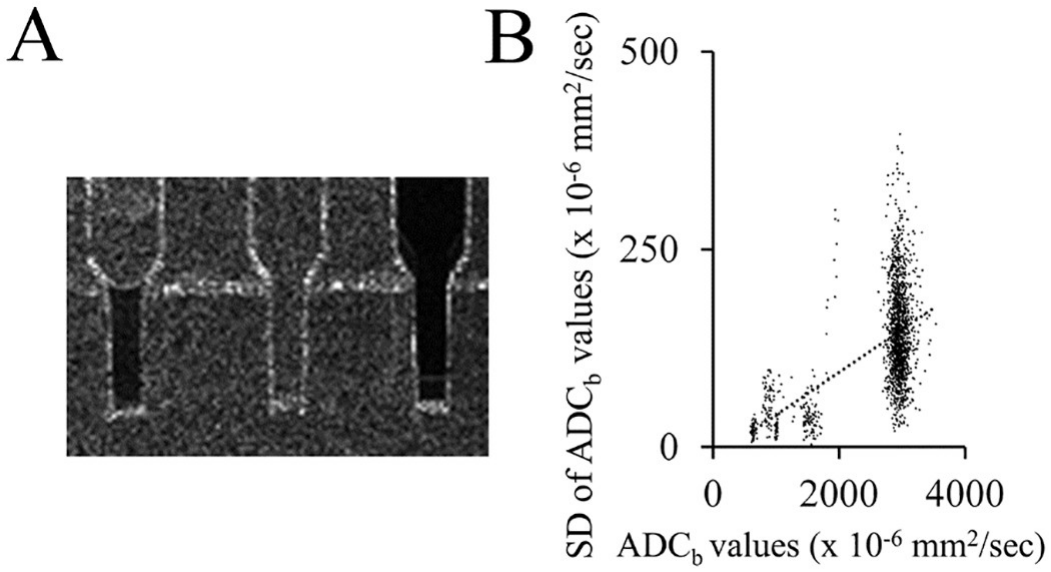
Relationship between ADC values and their SD. (A) SD image of the ADC_b_ values of different phantoms. High-cellularity bio-phantom, physiological saline phantom and 120 mM polyethylene glycol phantom were indicated from left to right. (B) Scatter graph indicating the correlation between the values of ADC_b_ and SD of ADC_b_. The dotted line indicates a positive correlation as a result of linear regression. ADC, apparent diffusion coefficient; ADC_b_, ADC basic; SD, standard deviation.

Fig 4A indicates SD images of the different phantoms. HC bio-phantom and the 120 mM PEG phantom show low signal intensity compared with the higher signal intensity of PS, exhibiting similarities in the respective ADC maps. Fig 4B shows quantitative correlation analysis between the values of ADC_b_ and the SD of ADC_b_. These values were determined from a 3.8x4.3 mm region of interest (ROI) of HC and PEG phantoms, and 13.9x14.5 mm and 3.8x4.3 mm ROIs of PS. A positive correlation is observed between the values, since the SD values increase when ADC_b_ values increase. Linear regression analysis resulted in the following equation: SD = 0.054 x ADC_b_ − 13.854, where the R^2^-value is 0.31.

### Quantitative evaluation of the DK image and ASM images

**Fig 5:**
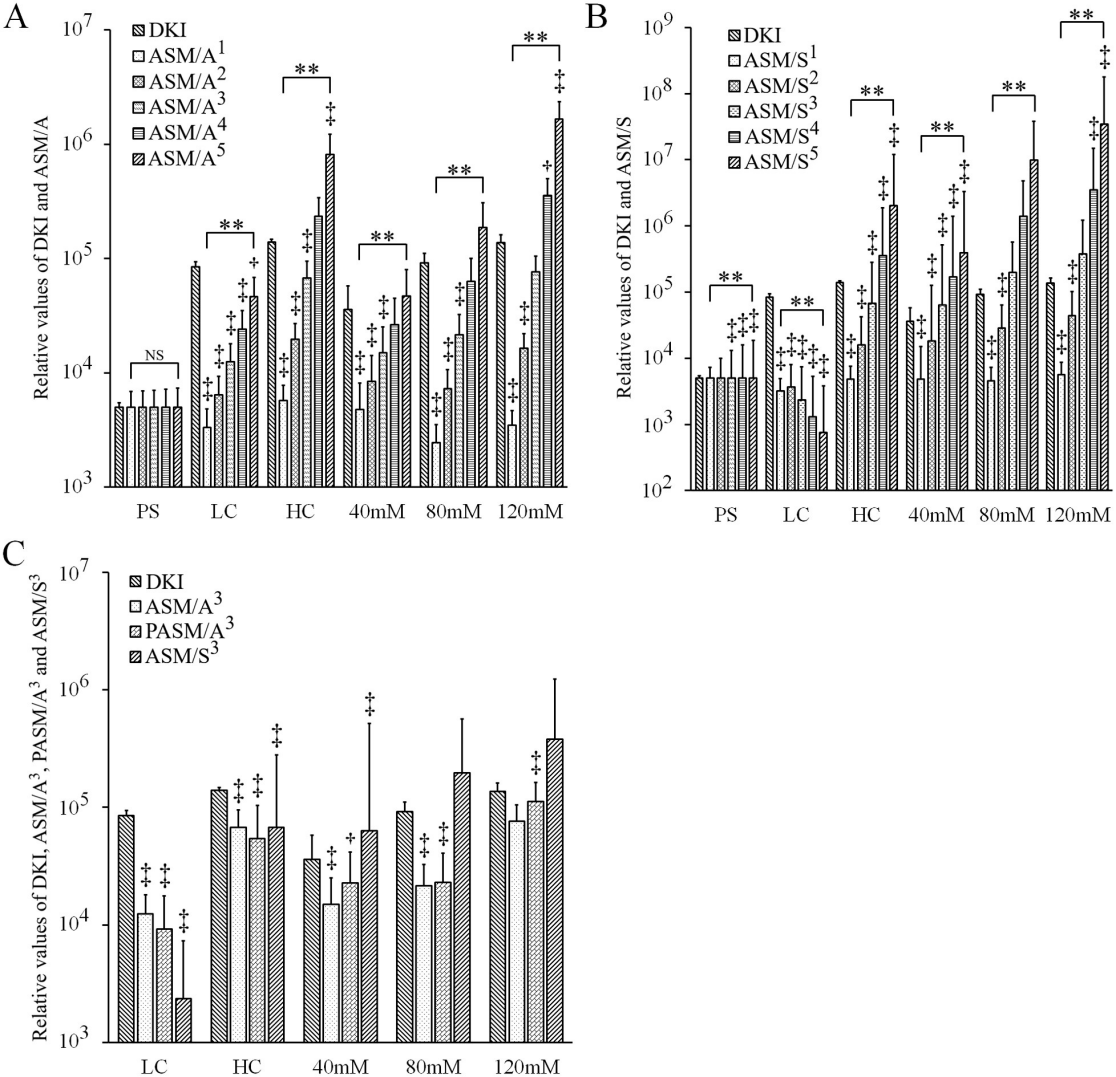
Comparison of the relative values for various phantoms among the DK image and ASM images. (A) ASM/A images. (B) ASM/S images. (C) ASM/A^3^, PASM/A^3^ and ASM/S^3^ images. Phantoms used were bio-phantoms of Jurkat cells and polyethylene glycol phantoms. Vertical bar represents relative values of DK image and ASM images, which were modified for their PS values to become 5,000. Error bar represents standard deviation for each value. ^+^P<0.05 and ^‡^P<0.01, Dunn’s method added to Kruskal-Wallis test for comparison between values of DK image and each ASM image. **P<0.01 and NS, not significant by Kruskal-Wallis test for comparison among the values of ASM images. ADC, apparent diffusion coefficient; ASM, ADC subtraction method; PS, physiological saline; LC, low-cellularity; HC, high-cellularity; DKI, diffusion kurtosis imaging; ASM/A^x^, ASM division by ADC x times; ASM/S^x^, ASM division by standard deviation x times; PASM/A^3^, positive ASM division by ADC three times.

The relative values of the DK image and ASM images were determined from a 3.8x4.3 mm ROI selected in six different phantoms, including PS, LC and HC bio-phantoms and 40 mM, 80 mM and 120 mM PEG phantoms. All relative values were modified for their PS values to become 5,000 as seen in Fig 5. Fig 5A shows a comparison of relative values among the DK image and ASM/A images. Among the ASM/A images, it was observed that when the number of divisions increases, the signal values also increase. Between DK image and each image of ASM/A, a significant difference (P<0.05 and P<0.01) is seen for all phantoms except for all of the PS phantom, the ASM/A^4^ of the HC bio-phantom, ASM/A^4^ and ASM/A^5^ of the 40 mM and 80 mM PEG phantoms, and ASM/A^3^ of the 120 mM PEG phantom. In addition, an increase in the SD values was noticed as a result of increasing the number of divisions. In spite of that, a significant difference was found among the five ASM/A images (P<0.01) for each phantom except for the PS phantom. The results of HC bio-phantom and 120 mM PEG phantom show a resemblance between DK imaging and the two types of ASM/A images (ASM/A^3^ and ASM/A^4^). In addition, compared with that in DK imaging, the ASM/A^5^ value appeared to be higher.

Fig 5B shows a comparison of relative values among the DK image and ASM/S images. The signal values increase when the number of divisions increases, except in the case of LC bio-phantom, which showed the opposite trend. A significant difference is observed between DK image and each image of ASM/S (P<0.05 or P<0.01) for each phantom except for ASM/S^1^ and ASM/S^2^ of the PS phantom, the ASM/S^3^, ASM/S^4^ and ASM/S^5^ images of the 80 mM PEG phantom and the ASM/S^3^ image of the 120 mM PEG phantom. Moreover, a significant difference was observed among the five ASM/S images (P<0.01) for each phantom.

Fig 5C exhibits a comparison between the DK image and the third division step of each ASM image (ASM/A^3^, PASM/A^3^ and ASM/S^3^ images). A significant difference is seen for each phantom (P<0.05 or P<0.01) except for the ASM/S^3^ image of the 80 mM PEG phantom and the ASM/A^3^ and ASM/S^3^ image of the 120 mM PEG phantom. Furthermore, no significant difference was observed between the original ASM/A^3^ and the positive value PASM/A^3^ for all phantoms except for that of the 120 mM PEG phantom (P<0.01). Concerning the concentration of PEG phantoms, the difference in ASM signal values within the high RD range between 80 mM and 120 mM PEG phantoms was bigger than the difference within the low RD range between 40 mM and 80 mM PEG phantoms for all ASM images. Within the low RD range, PASM/A^3^ showed almost no difference compared to ASM/A^3^ and ASM/S^3^ which showed small differences.

## Discussion

The present study provided insights on the potential of ASM/A image as one of the novel RD imaging techniques by using tumor-mimicking phantoms, specifically the HC bio-phantom and 120 mM PEG phantom. The results demonstrated the resemblance between DK imaging and the two types of ASM/A images (ASM/A^3^ and ASM/A^4^). In addition, it was revealed that compared with that in DK imaging, ASM/A^5^ is significantly more sensitive for RD, indicating its potential as a diagnostic tool for RD.

DW imaging is extensively applied as a non-invasive MR technique for the early detection of various diseases [21]. A DW imaging-derived ADC map is frequently used clinically [2]. It reflects the diffusion of water molecules within tissues that is affected by the presence or absence of barriers, including cell membranes and intracellular organelles [7]. Free diffusion represents the normal distribution of water molecules in the absence of barriers, whilst RD represents the limitation of movement by water molecules due to the presence of barriers [22].

The recently developed DK imaging technique is one RD imaging technique that is based on the principle of measuring the degree of non-Gaussian distribution of water molecule diffusion [23, 24]. It has been previously reported that the DK imaging technique has conferred high sensitivity and specificity for detecting tumors in the brain, kidney, liver and prostate, in addition to other diseases, including cerebrovascular infarction, Alzheimer’s disease, mild cognitive impairment, Parkinson’s disease, multiple sclerosis and aging-related changes in the brain microstructure [8, 25–28]. However, DK imaging has few limitations. A specific software is required to acquire DK images [29]. In addition, compared with other diffusion imaging methods, such as ADC maps, DK imaging requires longer imaging times since it requires a minimum of two non-zero b-values and ≥ three diffusion directions [23]. The need of multiple different b-values depends on the pathological conditions, which will result in different kurtosis values [1]. A fast DK imaging technique (Fast-DKI) has succeeded to shorten the long imaging time [30].

ASM has been recently developed as a new RD imaging technique based on a different principle to DK imaging. The ASM principle utilizes the difference in the ADC values in an image pair of two ADC maps (ADC_b_ and ADC_m_). The subtraction of the two ADC values via ASM, obtained using different EDT, might permit visualization of the diffusion phenomena in a narrow space of 1.0 μm [10]. ASM visualizes the sum of the signal values of restricted diffusion of hydrogen atoms on a 1.0 μm scale in each pixel with the size of individual pixels determined by the image resolution. To prepare ASM images for clinical use, an ASM imaging software was developed. This software completes the calculation process of ASM more efficiently and automatically through either a specific macro or a plugin written in Java language, both of which can be applied easily using the ImageJ program [11]. Additionally, to make the ASM more suitable and efficient for clinical application, a short imaging sequence with 3 min 41 sec for brain ASM imaging as an example, was developed [12], which is almost similar to that of Fast-DKI [30].

In terms of phantoms for RD, the PEG phantom has been reported to mimic the conditions of RD *in vitro*, indicating that it can be utilized as the standard phantom for RD imaging [13].

In the present study, a comparison was made between the ASM/A and ASM/S images. The difference of ADC values in an image pair of two ADC maps (ADC_b_ and ADC_m_) is affected by the error during the imaging of ADC maps. This error becomes amplified when the ADC value increases [10]. Therefore, resolving this error is necessary for the calculation process of ASM. Division by SD image was created for this purpose, however, SD imaging requires the repeated imaging of ADC_b_, which is relatively time consuming. For this reason, it is difficult to obtain SD images in the clinic. Since SD images share similarity with the ADC map, division by ADC_b_ instead of the SD image was used for ASM calculation. A comparison between ASM/A and ASM/S images showed the same tendency, where an increase in the number of divisions results in an increase in signal intensity in both ASM/A and ASM/S, suggesting that ASM/A could be used clinically without the need of long imaging times. However, further investigation is necessary to clarify the reason for the unusual discrepancy shown between ASM/A and ASM/S of LC bio-phantom.

In the principle of ASM, as ADC_m_ has a longer EDT compared with ADC_b_, only a positive value of the difference between ADC_m_ and ADC_b_ should appear. However, ideal positive values were affected by imaging error of ADC maps. Therefore, the use of the absolute difference value between ADC_b_ and ADC_m_ in ASM/A was proposed for ASM calculation processes [10] instead of the positive value approach, PASM/A. Eventually, a comparison between ASM/A and PASM/A was made and as a result, both images showed similarities, indicating the possibility of using ASM/A in the clinic.

As one of the limitations, ASM requires two ADC maps, which means longer imaging time. Even though a fast-imaging sequence of ASM has been recently developed [12], much faster imaging sequences should be developed for future clinical trials of ASM. Another limitation is that the present study evaluated the phantoms alone, which possess isotropic properties, without including lesions in human organs, some of which, such as the nervous system, have diffusion anisotropic properties. As a result, some discrepancies may appear in the future clinical trials for anisotropic lesions. To overcome these issues, clinical trials of ASM are required to check the accuracy of the calculation process of ASM. Moreover, for the current study, the maximum number of divisions performed during the calculation process of ASM/A is five divisions. The possibility of a more sensitive image for RD might be high after increasing the number of divisions beyond five times. Therefore, further basic studies are recommended to improve ASM techniques in order to apply them in the clinic.

In conclusion, a comparison among several ASM types was performed in the present study for the purpose of future clinical trials. ASM/A^3^ and ASM/A^4^ images were found to mimic the DK image. In addition, ASM/A^5^ image appeared to be more sensitive for RD compared with the DK image. These results indicate the potential of ASM/A images as a diagnostic tool for visualizing RD phenomena in the clinic as the same as the DK image. Future clinical trials are necessary to prove the different and unknown merits of both methods in the clinic.

## Supporting information

S1 Dataset Dataset for Fig 3. (XLSX)

S2 Dataset Dataset for Fig 4. (XLSX)

S3 Dataset Dataset for Fig 5. (XLSX)
